# Incidence, Predictive Factors and Long-Term Clinical Impact of Left Ventricular Remodeling According to the Completeness of Revascularization in Patients with ST-Elevation Myocardial Infarction and Multivessel Disease

**DOI:** 10.3390/jcm11216252

**Published:** 2022-10-23

**Authors:** Min Chul Kim, Yongwhan Lim, Youngkeun Ahn, Joon Ho Ahn, Seung Hun Lee, Dae Young Hyun, Kyung Hoon Cho, Doo Sun Sim, Young Joon Hong, Ju Han Kim, Myung Ho Jeong

**Affiliations:** Department of Cardiology, Chonnam National University School of Medicine, Chonnam National University Hospital, Gwangju 61469, Korea

**Keywords:** left ventricular remodeling, ST-segment elevation myocardial infarction, multivessel disease, complete revascularization, heart failure

## Abstract

In this study, we identified several factors related to left ventricular remodeling (LVR) and examined the impact of LVR on the prognosis of patients with ST-elevated myocardial infarction and multivessel disease treated with complete (CR) or incomplete (IR) revascularization. LVR was defined as an LV end-diastolic diameter >55 mm. A total of 262 patients without LVR at presentation were followed up with echocardiography between 1 month and 1 year. The primary outcome was a composite of all-cause death (AD), MI, and heart failure (HF), referred to as a major adverse cardiovascular endpoint (MACE). Then, each variable was analyzed as a secondary outcome. Follow-up echocardiography identified 26 patients (9.9%) with LVR. LVR was associated with an initial LV ejection fraction <50%, Killip 3 disease at presentation, and a peak troponin I level >70 mg/dL. Survival analysis showed an association between LVR and adverse outcomes only in the IR group, in which the adjusted hazard ratio (HR) was increased for the MACE (HR = 3.22, 95% confidence interval (CI) = 1.19–8.71, *p* = 0.002) and HF (HR = 21.37, 95% CI = 4.47–102.09, *p*< 0.001), but not for the CR group. In STEMI with MVD, LVR within the first year after percutaneous coronary intervention was associated with worse outcomes in the IR but not the CR group.

## 1. Introduction

Left ventricular remodeling (LVR) is a maladaptation of the heart to mechanical, neurohormonal, and inherited changes, with effects on ventricular size, shape, and function [1]. Compared to physiologic or adaptive remodeling, pathologic LVR leads to significant and disproportionately adverse outcomes after myocardial infarction (MI) [2,3].

Patients with ST-segment elevation myocardial infarction (STEMI) with significant stenosis in the nonculprit artery, and thus with multivessel disease (MVD), have a worse prognosis than their counterparts without MVD. The proportion of STEMI patients with MVD is >50% [4].

Although LVR after acute MI is a well-known prognostic factor, and despite the high rate of MVD in STEMI patients, few reports have provided a detailed assessment of LVR in this population [5]. LVR development with respect to the revascularization strategy thus remains poorly understood. In this study, we examined the relationship between LVR development and long-term prognosis according to the revascularization strategy in STEMI patients with MVD. Specifically, our aim was to identify the predictor(s) of LVR development in these patients and then determine the impact of LVR development during follow-up on the long-term prognosis according to the revascularization strategy: complete or incomplete revascularization (CR or IR).

## 2. Materials and Methods

### 2.1. Study Population

All patients with STEMI and MVD treated with percutaneous coronary intervention (PCI) at Chonnam National University Hospital between January 2006 and July 2009 were enrolled. A diagnosis of STEMI with a 12-lead electrocardiogram (ECG) was established based on the criteria suggested at the time of diagnosis and was confirmed based on coronary angiographic finding(s) and/or increased levels of cardiac-specific biomarkers [6].

**Figure 1:**
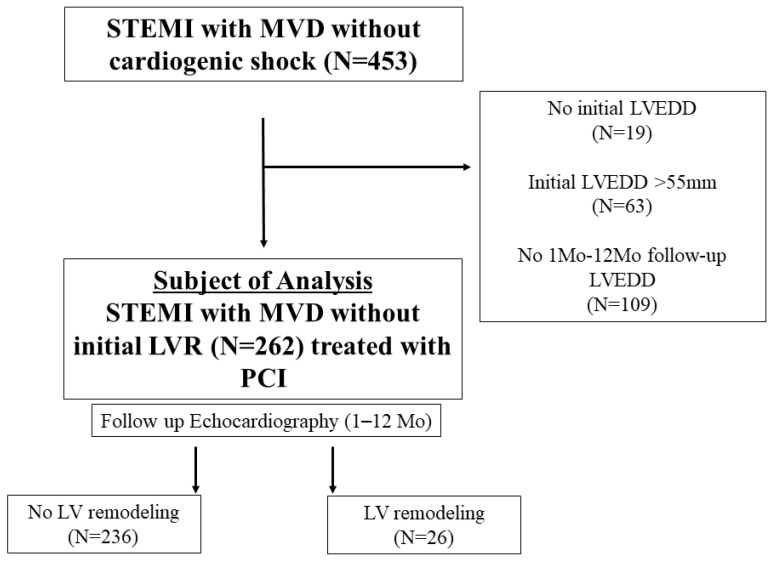
Study flow. LV= left ventricular; LVEDD= left ventricular end-diastolic diameter; LVR= left ventricular remodeling; MVD= multivessel disease; PCI= percutaneous coronary intervention; STEMI= ST segment elevation myocardial infarction.

Among 575 patients, after the exclusion of those with cardiogenic shock at presentation or who died before discharge (n = 122), 453 patients were identified. LVR was defined as an LV end-diastolic diameter (LVEDD) > 55 mm based on normal echocardiographic measurements in the Korean population [7]. After the exclusion of patients with LVR at presentation (n = 63), no information on LVEDD during the index hospitalization (n = 19), or no follow-up echocardiography or information on LVEDD between 1 month and 1 year after discharge from index hospitalization (n = 109) (Figure 1), 262 patients who did not have LVR at presentation and for whom follow-up echocardiography was performed between 1 month and 1 year were included in the analysis. All patients were over 20 years of age. The study protocol followed the Declaration of Helsinki in terms of investigations in humans and was approved by the Institutional Review Board of our institution (IRB number: CNUH-2017-129).

### 2.2. Treatment and Data Collection

Each patient’s medical history was obtained, and laboratory tests, except lipid profiles, were performed immediately after admission to the emergency department and before PCI. Lipid profiles were obtained after at least 9 h of fasting within 24 h of hospitalization. Echocardiography during index hospitalization was performed before or just after the index PCI for STEMI.

Patients scheduled for a PCI were given 300 mg aspirin and 600 mg clopidogrel, as loading doses, before PCI. Unfractionated heparin infusion at a dose of 50–70 U/kg was performed at the initiation of PCI, with an additional dose injected to maintain an activated clotting time of 250–300 s. After PCI, the patients received 100 mg aspirin and 75 mg clopidogrel.

Quantitative coronary analysis (QCA) was performed during the index PCI in all patients. The culprit artery was determined based on the ECG finding(s). A lesion in the left anterior descending, left circumflex artery, and right coronary artery was considered significant if it resulted in a ≥50% stenosis diameter. In the left main coronary artery, a stenosis diameter ≥30% was considered significant. The interventional strategy for a nonculprit artery(s), was at the operator’s discretion. PCI for a nonculprit artery(s) was performed either simultaneously with PCI of the culprit artery or as a staged procedure.

Patients underwent follow-up echocardiography between 1 and 12 months (median 6.66 months, (25th percentile/75th percentile) = 2.98 months/8.6 months), at which time the development of LVR was determined.

### 2.3. Study Definitions and Outcomes

LVR was defined as LVEDD > 55 mm regardless of the LV ejection fraction (EF). LVEDD was measured in M-mode tracing using the method recommended in most updated guidelines [8]. The cut-off for LVR using LVEDD was determined based on the cut-off data of South Koreans reported in a previous study [7]. CR was defined when total revascularization was performed for both the culprit and nonculprit artery(s) with a significant lesion(s), and IR was when there was a coronary artery with a significant remnant lesion.

The primary outcome was major adverse cardiovascular outcome (MACE), defined as a composite of all-cause death (AD), recurrent MI, and readmission due to heart failure (HF). Each of these variables was analyzed as a secondary outcome according to the presence of LVR and the revascularization strategy.

### 2.4. Statistical Analysis

Categorical variables were analyzed using the Chi-square or Fisher’s exact test. Continuous variables were analyzed using Student’s *t*-test or the Wilcoxon rank-sum test and are expressed as the mean ± SD or median and interquartile range. Paired data was compared using paired t-test or Wilcoxon singed rank test according to normality or sample size. *p*-values were two-tailed, with *p* <0.05 considered to indicate statistical significance.

Factors related to LVR were identified in univariable and multivariable logistic regression analyses. An ideal multivariable model was selected using a bidirectional approach.

Survival was analyzed for predefined outcomes. Kaplan–Meier curves were used to compare primary and secondary outcomes between LVR and non-LVR patients differing in their revascularization status: CR vs. IR.

Cox proportional hazards regression modeling was used to test whether the development of LVR during follow-up was an independent predictor of clinical outcome. The multivariable analysis consisted of variables determined to be significant in the univariable analysis (*p* < 0.1) and/or variables with a known effect on outcome, such as age >65 years, sex, hypertension, serum creatinine, hemoglobin, Killip class, peak troponin I level, discharge medication, including beta-blocker, angiotensin-converting enzyme (ACE) inhibitor or angiotensin II receptor blocker (ARB), statin use, hemoglobin A1C (HbA1C), and N-terminal pro-brain natriuretic peptide (NT-proBNP) level. A bidirectional or forward approach was used to select the ideal Cox proportional hazard model.

The effects of confounding factors with different distributions between LVR and non-LVR patients were minimized by calculating the propensity score (PS) and using inverse probability treatment weighting (IPTW). The PS was calculated using the following variables: age, sex, systolic blood pressure, diastolic blood pressure, heart rate at presentation, Killip class, previous coronary artery disease, hypertension, diabetes mellitus, history of smoking, previous cerebrovascular accident, initial LVEF, initial LVEDD, hemoglobin, serum creatinine, CK-MB, peak troponin I, NT-proBNP, low-density lipoprotein cholesterol (LDL-C), high-density lipoprotein cholesterol (HDL-C), HbA1C, discharge medication, including beta-blocker, ACE inhibitor or ARB, statin use, and number of diseased vessels. The PS and IPTW were calculated using the toolkit for the weighting and analysis of nonequivalent groups (TWANG) package. Balancing before and after weighting is described in Supplementary Figures S1–S3 and Tables S1–S3.

All statistical analyses were performed using the R statistical package (version 4.2.0; R Foundation for Statistical Computing, Vienna, Austria. https://www.R-project.org, accessed on 20 October 2022).

## 3. Results

Among the 262 patients with STEMI and MVD, LVR developed after PCI in 26 patients (9.9%), as determined via follow-up echocardiography. The 236 patients without LVR were compared with these 26 patients with LVR. The incidence of LVR between the CR and IR groups was not statistically significant (8.9% vs. 11.2%, *p* = 0.681).

### 3.1. Baseline, Therapeutic, and Angiographic Characteristics

The baseline and laboratory characteristics of the LVR and non-LVR groups are described in Table 1.

Patients who developed LVR more frequently presented with Killip 3 classification (4.2% vs. 19.2%, *p* < 0.007) and had higher peak troponin-I levels (median: 41.6 mg/dL vs. 2.1 mg/dL, *p* = 0.032). The level of NT-proBNP in the LVR and non-LVR groups was not statistically different (216.0 pg/mL vs. 310.5 mg/mL, *p* = 0.789). At initial echocardiography, patients who subsequently developed LVR had a lower LVEF (57.8 ± 10.4 vs. 47.3 ± 10.5, *p* < 0.001) and a larger LVEDD (median: 49 mm vs. 53.0 mm, *p* = 0.001).

The angiographic and therapeutic characteristics of the patients are described in Table 2.

Door-to-balloon time was not significantly different in the LVR and non-LVR groups (78.0 min vs. 83.5 min, *p* = 0.279). The culprit artery distribution and the proportions of two- and three-vessel disease were comparable. All or nearly all patients were treated with PCI and a stent (99.2% vs. 100%, *p* = 1). The rates of multivessel PCI (71.6% vs. 65.4%, *p* = 0.663) and CR (56.4% vs. 50.0%, *p* = 0.681) were also comparable. The rates of acute kidney injury, atrioventricular block, fatal arrhythmia, and temporary pacemaker and intra-aortic balloon pump use in LVR and non-LVR patients were not statistically different. The prescription rate of discharge medication was also statistically comparable.

### 3.2. Echocardiographic Data

**Figure 2:**
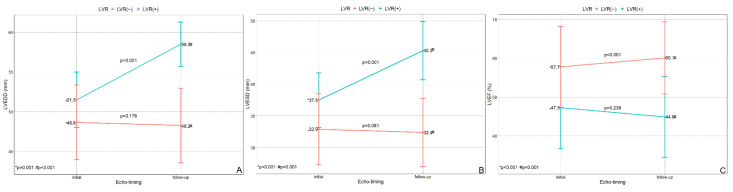
Echocardiographic data at both initial and follow-up echocardiography (performed in 6.66 months as median after index PCI) according to left ventricular remodeling. (**A**) LVEDD, (**B**) LVESD, and (**C**) LVEFLVEDD. LVEDD = left ventricular end-diastolic diameter; LVEF = left ventricular ejection fraction; LVESD = left ventricular end-systolic diameter; LVR = left ventricular remodeling.

Echocardiographic data are presented in Figure 2. Compared to patients without LVR, initial LVEDD was larger in patients with LVR (48.7 ± 4.7 mm vs. 51.5 ± 3.5 mm, *p* = 0.001) as was the initial LVESD (33 ± 5.6 mm vs. 37.5 ± 4.3 mm, *p* < 0.001). LVEF was larger in patients without than with LVR (57.7 ± 10.4% vs. 47.3 ± 10.5%, *p* < 0.001).

At follow-up echocardiography (performed at a median 6.66 months after index PCI), compared to patients without LVR, those with LVR had a larger LVEDD (48.3 ± 4.7 mm vs. 58.5 ± 2.8 mm, *p* < 0.001) and LVESD (32.4 ± 5.4 mm vs. 45.3 ± 4.6 mm, *p* < 0.001), and a smaller LVEF (60.1 ± 9.3% vs. 45.0 ± 10.4%, *p* < 0.001).

In patients without LVR, LVEDD and LVESD did not change significantly. LVEF, however, increased (from 57.7 ± 10.4% to 60.1 ± 9.3%, *p* < 0.001). Conversely, in patients with LVR, both LVEDD (from 51.5 ± 3.5 mm to 58.5 ± 2.8 mm, *p* < 0.001) and LVESD (from 37.5 ± 4.3 mm to 45.3 ± 4.6 mm, *p* < 0.001) increased. The change of LVEF in patients with LVR was not statistically significant (*p* = 0.238).

### 3.3. Factors Related to LVR

Logistic regression was performed to identify the factors related to LVR after index PCI in patients with STEMI and MVD. The results are described in Table 3.

In the univariable analysis, LVEF < 50%, LVEDD > 50 mm at initial echocardiography, Killip 3 disease, a peak troponin I level > 70 mg/dL, and an NT-proBNP level > 500 pg/mL at presentation were related to LVR, based on an increased odds ratio (OR). In the multivariable analysis after backward model adjustment, LVEF < 50% (OR = 3.62, 95% confidence interval (CI) = 1.45–9.07, *p* = 0.006), Killip 3 disease (OR = 4.89, 95% CI = 1.31–18.30, *p* = 0.018), and a peak troponin I level > 70 mg/dL (OR = 2.79, 95% CI = 1.09–7.17, *p* = 0.032) were factors that increased the OR for LVR.

### 3.4. Outcomes According to LVR and PCI Strategy and Survival Analysis

Outcomes according to LVR and the PCI strategy are described in Table 4. The median follow-up duration was 6.52 years (25th percentile and 75th percentile = 4.39 years and 7.89 years, maximum: 9.59 years).

Overall, compared to patients without LVR, those with LVR had higher rates of MACE (12.7% vs. 34.6%, *p* = 0.007) and readmission due to HF (5.1% vs. 26.9%, *p* < 0.001). In patients with CR, the incidence of all outcomes, including MACE, AD, MI, and HF, was not significantly different compared to either group. However, patients with IR and LVR had a higher incidence of MACE (17.5% vs. 53.8%. *p* = 0.008) and HF (3.9% vs. 46.2%, *p* = 0.001).

**Figure 3:**
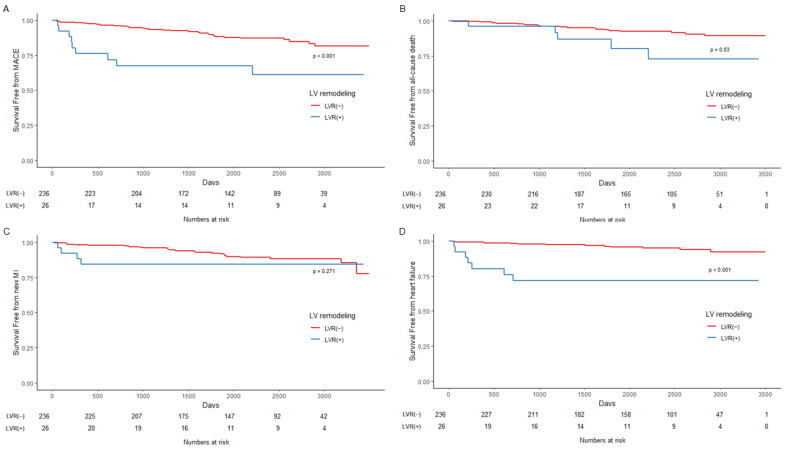
Kaplan–Meier curves for outcomes. (**A**) Composite outcome of all-cause death, myocardial infarction, and readmission due to heart failure, thus MACE; (**B**) All-cause death; (**C**) Myocardial infarction; (**D**) Readmission due to heart failure. LVR = left ventricular remodeling; MACE = major adverse cardiovascular outcome; MI = myocardial infarction.

**Figure 4:**
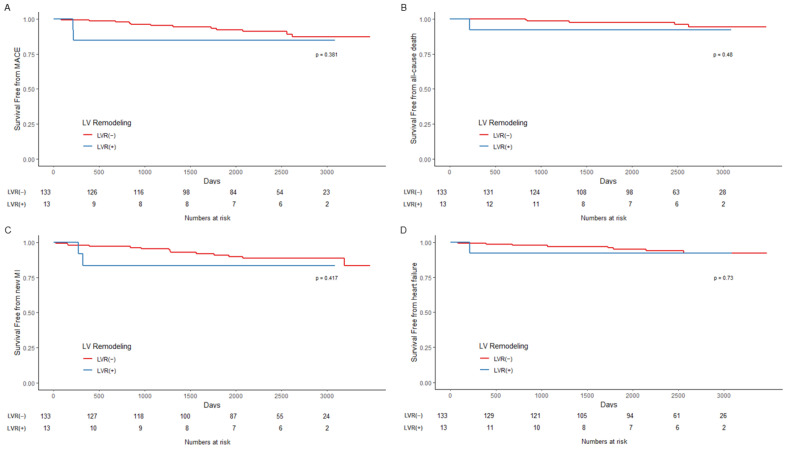
Kaplan–Meier curves for outcomes in patients who had complete revascularization. (**A**) Composite outcome of all-cause death, myocardial infarction, and readmission due to heart failure, thus MACE; (**B**) All-cause death; (**C**) Myocardial infarction; (**D**) Readmission due to heart failure. LVR = left ventricular remodeling; MACE = major adverse cardiovascular outcome; MI = myocardial infarction.

**Figure 5:**
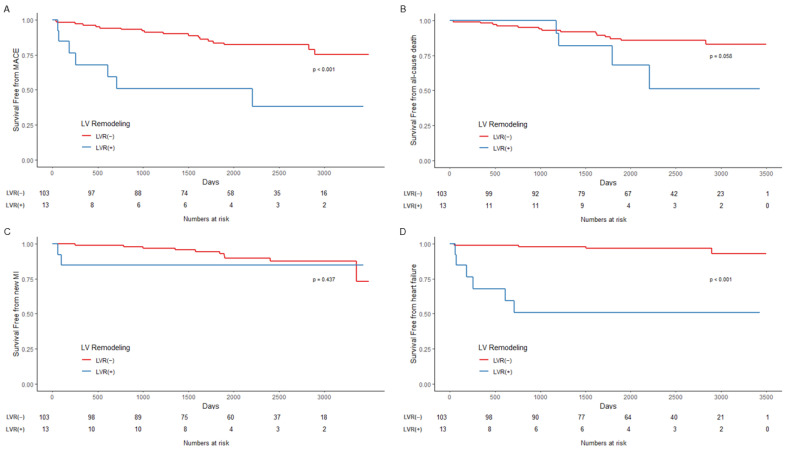
Kaplan–Meier curves for outcomes in patients who had incomplete revascularization. (**A**) Composite outcome of all-cause death, myocardial infarction, and readmission due to heart failure, thus MACE; (**B**) All-cause death; (**C**) Myocardial infarction; (**D**) Readmission due to heart failure. LVR = left ventricular remodeling; MACE = major adverse cardiovascular outcome; MI = myocardial infarction.

The results of survival analysis are described in Figure 3, Figure 4 and Figure 5 and Table 5. In the Kaplan–Meier curve for overall outcomes, patients with LVR had a higher probability of MACE (*p* < 0.001), AD (*p* = 0.03), and readmission due to HF (*p* < 0.001) throughout the follow-up period (Figure 3). In patients with CR, the probability of all outcomes was not statistically different from that of patients with LVR (Figure 4). However, patients with LVR and IR had a higher probability of MACE (*p* < 0.001) and readmission due to HF (*p* < 0.001) (Figure 5).

The results of Cox proportional modeling and the HR according to LVR development and outcome are provided in Table 5. The corresponding tables (Tables S1–S3) and figures (Figures S1–S3) showing the results before and after IPTW are provided in the Supplementary Materials. Overall, the adjusted HR was not significant except for readmission due to HF (adjusted HR = 4.73, 95% CI = 1.57–11.63, *p* = 0.004), although this relationship was not maintained in the modeling with IPTW (IPTW adjusted HR = 2.39, 95% CI = 0.86–6.60, *p* = 0.09). According to the Cox proportional modeling, in the CR group, LVR was not significantly related to an increased HR for all outcomes. In the IR group, however, LVR was associated with an increased HR for MACE (adjusted HR = 3.51, 95% CI = 1.34–9.18, *p* = 0.010) and HF (adjusted HR = 23.65, 95% CI = 5.64–99.06, *p* < 0.001). These associations were maintained after IPTW-adjusted modeling.

## 4. Discussion

In the first follow-up echocardiography after PCI for STEMI with MVD, 9.9% of the patients had newly developed LVR, defined as LVEDD > 55 mm. According to the echocardiographic data, patients with LVR had a larger LVEDD and LVESD and a smaller LVEF than those without LVR at both initial and follow-up echocardiography. The risk factors for LVR development after PCI were initial LVEF < 50%, Killip 3 disease at presentation, and a peak troponin I level > 70 mg/dL. The completeness of revascularization did not affect the development of LVR after PCI. Finally, the survival analysis showed the association of LVR with adverse outcomes, including MACE and readmission due to HF, only in the IR group but not the CR group.

### 4.1. Definition and Incidence of LVR

According to previous reports, the incidence of LVR at 1 year after STEMI ranges from 30% to 48% [3,5,9,10]. The development of LVR after revascularization seems to have a variable time course and is more frequent during the first 3 months after STEMI [3], but it may be progressive [2,3,5]. In our study, the incidence of LVR (9.9%) was lower than in previous reports.

Possible explanations for this difference include a difference in the definition of LVR. Echocardiography is a standard and first-line imaging modality used to detect and define LVR [1]. Most clinicians have adopted a 20% increase in left ventricular end-diastolic volume (LVEDV) as the echocardiographic definition of LVR, as this value is considered to reflect the maladaptive changes in LV size [2,3,5,9]. The use of cardiac magnetic resonance imaging (CMR) to define LVR is controversial, and different cut-off values have been applied [10,11,12]. The definition of LVR used in our study, LVEDD > 55 mm, was determined based on a previous study of a South Korean population [7]. Because echocardiographically determined LVEDD correlates well with LVEDV [13], this definition presumably reflects an LVEDV above the normal range. In addition to the difference in the parameters used to define LVR (LVEDD vs. LVEDV), the adoption of an absolute cut-off value (LVEDD > 55 mm) rather than a relative change (%) as the criterion defining LVR may have accounted for the lower rate of LVR in our patients. These differences in the definition of LVR also complicate comparisons between our study and previous ones.

Moreover, the timing of the echocardiographic follow-up may influence the incidence of LVR. Although a previous study suggested that LVR frequently develops within the first 3 months after STEMI, in a significant proportion of patients it develops later [3,5]. In our study, the median echocardiographic follow-up period was 6.6 months; this <1-year follow-up period could explain the lower incidence of LVR.

Despite these considerations, the definition used in this study was adopted because of its simplicity, as it was based on a single parameter determined in the studied population. Its predictive value for outcomes after PCI in STEMI and MVD is discussed below.

### 4.2. Risk Factors for LVR Development

Infarct size is one of the most important predictors of LVR after revascularization in STEMI [14,15]. In those cited studies, infarct size was measured using CMR, with larger infarct size [14,15] and/or microvascular obstruction, as well as the transmurality of the infarct [14] related to LVR development. In our study, although CMR was not used to directly measure infarct size after revascularization, a peak troponin I level >70 mg/dL was associated with LVR development. This association was also reported in a study with a larger sample size [3].

We also identified initial LVEF < 50% as another risk factor for LVR development. A previous study found a relationship between infarct size and initial echocardiographic LVEF [16] and peak troponin concentration [17,18] in STEMI after revascularization. Both risk factors identified in our study, a peak troponin I level > 70 mg/dL and initial LVEF < 50%, could reflect infarct size.

Another risk factor for LVR development identified in our analysis was Killip classification 3 disease. In AMI, the Killip classification is a prognostic factor for both short- and long-term mortality [19,20]. A retrospective study suggested that Killip classification 1 or 2 predicts a recovery of LV systolic function after revascularization in AMI patients with depressed LV systolic unction (EF < 45%) [21]. Despite further evidence of a correlation between Killip classification and LV systolic function [22], many other factors are related to the Killip classification at presentation in STEMI [23]. Studies that have used a serial change in LVED as a definition of LVR either did not find any difference in the Killip classification [3] of LVR vs. non-LVR patients or did not include information on the Killip classification [2,5]. Our observation of an association between Killip classification 3 at presentation and LVR development thus merits further evaluation.

In our analysis, the completeness of revascularization in STEMI and MVD did not affect the development of LVR. Current guidelines recommend the revascularization of the significant noninfarct-related artery in AMI [24,25], based on the observation of many studies showing the benefit of CR in this population. In pooled analyses, the CR of the significant noninfarct-related artery(s) patients with STEMI with MVD was associated with lower rates of future revascularization [26], MI [27], and cardiovascular death [28,29]. In terms of LVR development, however, the impact of CR in patients with STEMI with MVD has not been well studied. In a randomized trial in which patients underwent CMR 3 months after index PCI, there was no difference in LVR development, in terms of a change in LVEDV, LVESV, and infarct size between the culprit-only and fractional flow reserve (FFR)-guided CR groups [30]. Another work also found no difference in LVR development between the culprit-artery-only PCI and preventive PCI groups [31]. Our findings are consistent with those findings in that the development of LVR after PCI for STEMI and MVD was related to infarct size itself rather than to the completeness of revascularization for nonculprit arteries with significant lesion(s). Further prospective studies are necessary to elucidate the relationship between the completeness of revascularization and LVR development in STEMI and MVD.

### 4.3. LVR and the Completeness of Revascularization: Impact on Long-Term Clinical Outcomes in STEMI with MVD

We found a negative impact of LVR development at 6 months on the long-term outcomes of patients who underwent PCI for STEMI with MVD. In our analysis, this group had a higher incidence of adverse composite outcome and readmission due to HF. This finding is consistent with previous reports published in the modern reperfusion era that have reported an association between LVR after PCI for STEMI and a higher incidence of HF [2,3].

Another major finding of our study was that the adverse composite outcome and readmission due to HF were associated with LVR development only in patients with IR in nonculprit arteries. This is a novel finding, given that most previous studies of LVR development after STEMI reperfusion have included patients regardless of MVD status [2,3,11,12], while studies that have examined the benefit of CR after STEMI and MVD have not focused on LVR development according to the completeness of revascularization. The difference in the impact of LVR on long-term outcomes according to the completeness of revascularization should be further investigated. We divided patients with LVR into CR and IR groups (Tables S2 and S3). Patients in those groups who developed LVR had similar characteristics, including lower initial LVEF and larger LVEDD, and a higher peak troponin I level, with a statistically significant difference only in the CR group. However, an association between LVR development and worse long-term clinical outcomes (MACE and readmission due to HF) was found only in the IR group in multivariable Cox proportional modeling, and it was maintained after IPTW adjustment for multiple variables. Our finding suggests a protective effect of preventive PCI of the nonculprit artery in patients with STEMI and MVD at risk of developing LVR. The impact of LVR development according to revascularization strategy in patients with STEMI with MVD should be investigated in a prospectively designed study. A further topic of interest is CR in patients who develop LVR during the early period after culprit-artery-only PCI.

### 4.4. Limitations

Our study had several limitations. First, the analysis was performed at a single-center using a retrospective database from which a limited number of patients were determined to be eligible. The criterion for LVR development (LVEDD > 55 mm) resulted in a lower incidence of LVR development than previously reported. Because of this lower incidence (~10%), and the small number of patients with LVR (26 patients), a strict statistical adjustment of the variables was not possible. Furthermore, revascularization of the nonculprit artery was assessed at the operator’s discretion, without the evaluation of other parameters such as FFR. In addition, follow-up echocardiography was performed at a median of 6.6 months after index PCI, which was sooner than in similar studies. Last, patients as the subject for analysis were enrolled from 2006 to 2009, which is a long time from the time of analysis. Although our purpose was to evaluate the long-term impact of LVR occurring early after PCI, this topic could be handled with more recent data, including with the advanced features of patient management that have improved over time.

## 5. Conclusions

LVR development, defined as an LVEDD > 55 mm, was detected in ~10% of our study patients. Risk factors for LVR development were an initial LVEF < 50%, a peak troponin I level > 70 mg/dL, and Killip 3 disease at presentation. LVR development was associated with higher risk for an adverse composite outcome and HF readmission only in the IR group of patients with STEMI with MVD.

## Figures and Tables

**Table 1:** Baseline characteristics according to LV remodeling.

	No LV Remodeling (N = 236)	LV Remodeling (N = 26)	*p* Value
Age	68.0 ± 11.9	71.5 ± 10.1	0.157
Male	173 (73.3%)	18 (69.2%)	0.833
SBP (mmHg)	130.0 [120.0; 150.0]	130.0 [120.0; 140.0]	0.187
DBP (mmHg)	80.0 [80.0; 90.0]	80.0 [70.0; 90.0]	0.21
HR (/min)	72.0 [64.0; 80.0]	79.0 [68.0; 88.0]	0.089
Killip 1	197 (83.5%)	20 (76.9%)	0.571
Killip 2	29 (12.3%)	1 (3.8%)	0.338
Killip 3	10 (4.2%)	5 (19.2%)	0.007
Previous CAD	16 (6.8%)	3 (11.5%)	0.624
Hypertension	112 (47.5%)	11 (42.3%)	0.77
DM	67 (28.4%)	11 (42.3%)	0.212
Smoking history	149 (63.1%)	16 (61.5%)	1
Previous CVA	8 (3.4%)	3 (11.5%)	0.147
LVEF (%)	57.8 ± 10.4	47.3 ± 10.5	<0.001
LVEDD (mm)	49.0 [46.0; 52.0]	53.0 [50.0; 54.0]	0.001
Hemoglobin (mg/dL)	13.1 [10.3; 14.8]	13.3 [12.6; 14.8]	0.515
Glucose (mg/dL)	155.0 [120.5; 187.0]	162.5 [129.0; 197.0]	0.592
Creatinine (mg/dL)	0.9 [ 0.8; 1.1]	0.9 [ 0.8; 1.0]	0.665
CK-MB (ng/mL)	55.9 [18.1; 109.2]	89.2 [33.2; 169.3]	0.06
Peak Troponin-I (mg/dL)	41.6 [16.3; 75.5]	72.0 [28.1; 119.8]	0.032
NT-proBNP (pg/mL)	216.0 [73.0; 511.0]	310.5 [62.0; 632.0]	0.789
Total cholesterol (mg/dL)	186.0 [161.0; 214.0]	172.5 [155.0; 214.0]	0.276
LDL cholesterol (mg/dL)	124.5 [103.0; 146.0]	119.0 [103.0; 143.0]	0.607
HDL Cholesterol(mg/dL)	44.0 [38.0; 52.0]	39.5 [34.0; 50.0]	0.064
Triglyceride (mg/dL)	106.0 [76.0; 142.0]	81.5 [61.0; 132.0]	0.034

Values are mean ± SD or median (25 percentile, 75 percentiles) according to distribution. BNP = brain natriuretic peptide, CAD = coronary artery disease, CVA = cerebrovascular accident; DBP = diastolic blood pressure; DM = diabetes mellitus; HR = heart rate; HDL = high-density lipoprotein; LDL = low-density lipoprotein; LVEDD = left ventricular end-diastolic diameter; LVEF = left ventricular ejection fraction; NT = N-terminal.

**Table 2:** Angiographic and therapeutic characteristics.

	No LV Remodeling (N = 236)	LV Remodeling (N = 26)	*p* Value
Door to balloon time (mins)	78.0 [61.0;92.0]	83.5 [53.0; 101.0]	0.279
Culpri			0.739
LM	2 (0.8%)	0 (0.0%)	
LAD	95 (40.3%)	13 (50.0%)	
LCX	25 (10.6%)	3 (11.5%)	
RCA	114 (48.3%)	10 (38.5%)	
Two-vessel disease	156 (66.1%)	17 (65.4%)	1
Three-vessel disease	80 (33.9%)	9 (34.6%)	1
Lesion types of a culprit lesion			0.121
Type B1	50 (21.2%)	9 (34.6%)	
Type B2	121 (51.3%)	14 (53.8%)	
Type C	65 (27.5%)	3 (11.5%)	
PCI			
PCI using stent(s)	234 (99.2%)	26 (100.0%)	1
Thrombus aspiration	15 (6.4%)	2 (7.7%)	1
TIMI 3 flow after PCI for a culprit artery	235 (99.6%)	25 (96.2%)	0.474
Multivessel PCI	169 (71.6%)	17 (65.4%)	0.663
Staged PCI	133 (56.4%)	17 (65.4%)	0.5
CR	133 (56.4%)	13 (50.0%)	0.681
Complications during hospitalization			
AKI	2 (0.8%)	0 (0.0%)	1
AV block	12 (5.1%)	1 (3.8%)	1
VF or Pulseless VT	8 (3.4%)	0 (0.0%)	0.724
TPM	19 (8.1%)	2 (7.7%)	1
IABP	5 (2.1%)	0 (0.0%)	1
Discharge medication			
Aspirin	236(100%)	26(100%)	
Clopidogrel	235 (99.6%)	26 (100.0%)	1
Cilostazole	149 (63.1%)	15 (57.7%)	0.741
Beta-blocker	210 (89.0%)	22 (84.6%)	0.734
ACE inhibitor or ARB	186 (78.8%)	21 (80.8%)	1
Statin	200 (85.1%)	20 (76.9%)	0.421
Spironolactone	19 (8.2%)	6 (23.1%)	0.038
Medication at 1 y			
Beta-blocker	167 (78.8%)	19 (76.0%)	0.951
ACE inhibitor or ARB	183 (86.3%)	22 (88.0%)	1.000
Spironolactone	10 (4.7%)	6 (24.0%)	0.001
Statin	186 (87.7%)	20 (80.0%)	0.440

Values are mean ± SD or median [25 percentile, 75 percentiles] according to distribution. AKI = acute kidney injury; ACE = angiotensin converting-enzyme; ARB = angiotensin receptor blocker; AV = atrioventricular; CR = complete revascularization; IABP = intra-aortic balloon pump; LAD = left anterior descending; LCX = left circumflex artery; LM = left main; PCI = percutaneous coronary intervention; RCA = right coronary artery; TIMI = thrombolysis in myocardial infarction; TPM = temporary pacemaker; VF = ventricular fibrillation; VT = ventricular tachycardia.

**Table 3:** Logistic regression: Factors related to LV remodeling.

Factors	Univariable		Multivariable	
	Unadjusted OR (95% CI)	*p* Value	Adjusted OR (95% CI)	*p* Value
Age > 65	2.05 (0.80–5.31)	0.137		
Male	0.82 (0.34–1.98)	0.657		
Initial LVEF < 50%	5.20 (2.25–12.04)	<0.001	3.62 (1.45–9.07)	0.006
LVEDD > 50 mm	3.34 (1.40–7.99)	0.006	2.47 (0.97–6.26)	0.056
Killip 2	0.29 (0.04–2.19)	0.227		
Killip 3	5.38 (1.68–17.21)	0.004	4.89 (1.31–18.30)	0.018
Hypertension	0.81 (0.36–1.84)	0.618		
DM	1.85 (0.81–4.23)	0.145		
Creatinine > 1.5 mg/dL	1.32 (0.28–6.17)	0.722		
Hemoglobin < 10 (mg/dL)	0.43 (0.12–1.48)	0.181		
Beta-blocker at discharge	0.68 (0.22–2.13)	0.509		
ACE inhibitor or ARB at discharge	1.13 (0.41–3.14)	0.816		
Statin at discharge	0.58(0.22–1.55)	0.276		
LAD or LM as a culprit	1.43 (0.64–3.23)	0.384		
Three-vessel disease	1.03 (0.44–2.42)	0.941		
Complete revascularization	0.77 (0.34–1.74)	0.536		
Peak Troponin I > 70 mg/dL	2.58 (1.13–5.85)	0.023	2.79 (1.09–7.17)	0.032
LDL cholesterol > 100 mg/dL	1.06 (0.41–2.77)	0.903		
NT pro-BNP > 400 pg/mL	3.04 (1.33–6.95)	0.008	2.40 (0.96–6.00)	0.061
HbA1c > 8%	2.01 (0.70–5.80)	0.196		

CI = confidence interval; DM = diabetes mellitus; LVEDD = left ventricular end diastolic diameter; LVEF = left ventricular ejection fraction; OR = odds ratio.

**Table 4:** Outcomes according to LV remodeling and PCI strategies.

Total			
	No LV remodeling (N = 236)	LV remodeling (N = 26)	*p* value
MACE	30 (12.7%)	9 (34.6%)	0.007
AD	19 (8.1%)	5 (19.2%)	0.129
MI	24 (10.2%)	4 (15.4%)	0.629
HF	12 (5.1%)	7 (26.9%)	<0.001
Complete revascularization			
	No LV remodeling (N = 133)	LV remodeling (N = 13)	*p* value
MACE	12 (9.0%)	2 (15.4%)	0.803
AD	5 (3.8%)	1 (7.7%)	1
MI	14 (10.5%)	2 (15.4%)	0.944
HF	8 (6.0%)	1 (7.7%)	1
Incomplete revascularization			
	No LV remodeling (N = 103)	LV remodeling (N = 13)	*p* value
MACE	18 (17.5%)	7 (53.8%)	0.008
AD	14 (13.6%)	4 (30.8%)	0.228
MI	10 (9.7%)	2 (15.4%)	0.881
HF	4 (3.9%)	6 (46.2%)	0.001

AD = all-cause death; HF = heart failure; LV = left ventricular; MACE = major adverse cardiac event; MI = myocardial infarction; PCI = percutaneous coronary intervention.

**Table 5:** Cox proportional modeling and HR of LV remodeling in total population, complete revascularization, and incomplete revascularization group.

Total						
	Unadjusted HR (95% CI)	*p* value	Adjusted HR (95% CI)	*p* value	IPTW-Adjusted HR (95% CI)	*p* value
MACE	3.54 (1.68–7.47)	0.001	2.09 (0.86–5.08)	0.10	2.19 (0.96–4.96)	0.059
AD	2.84 (1.06–7.61)	0.038	1.95 (0.71–5.32)	0.191	1.66 (0.72–3.83)	0.23
MI	1.80 (0.62–5.20)	0.278	1.68 (0.55–5.06)	0.356	1.41 (0.42–4.69)	0.569
HF	6.66 (2.62–16.95)	<0.001	3.98 (1.44–11.02)	0.004	2.61 (0.95–7.11)	0.06
Complete revascularization						
	Unadjusted HR (95% CI)	*p* value	Adjusted HR (95% CI)	*p* value	IPTW-adjusted HR (95% CI)	*p* value
MACE	1.93 (0.43–8.68)	0.39	1.79 (0.39–8.21)	0.44	1.26 (0.27–5.89)	0.76
AD	2.13 (0.25–18.26)	0.49	0.86 (0.09–8.14)	0.89	0.73 (0.068–8.58)	0.80
MI	1.84 (0.41–8.14)	0.424	1.33(0.27–6.33)	0.77	1.19 (0.34–4.08)	0.71
HF	1.44 (0.18–11.53)	0.732	0.98 (0.11–8.59)	0.99	0.80 (0.10–6.00)	0.83
Incomplete revascularization						
	Unadjusted HR (95% CI)	*p* value	Adjusted HR (95% CI)	*p* value	IPTW-adjusted HR (95% CI)	*p* value
MACE	4.49 (1.87–10.80)	0.001	3.51 (1.34–9.18)	0.01	3.22 (1.19–8.71)	0.02
AD	2.81 (0.92–8.56)	0.07	1.91 (0.58–6.25)	0.33	2.09 (0.66–6.58)	0.20
MI	1.83 (0.39–8.59)	0.44	1.66 (0.31–8.83)	0.55	1.71 (0.32–8.99)	0.52
HF	16.59 (4.64–59.29)	<0.001	23.65 (5.64–99.06)	<0.001	18.98 (4.79–75.10)	<0.001

AD = all-cause death; HF = heart failure; LV = left ventricular; MACE = major adverse cardiac event; MI = myocardial infarction.

## Data Availability

Not applicable.

## Supplementary Materials

The following supporting information can be downloaded at: https://www.mdpi.com/article/10.3390/jcm11216252/s1, Figure S1. Standardized effect size plot for estimating propensity scores in all patients. ES = effect size; KS = Komogorov-Smirnov. Figure S2. Standardized effect size plot for estimating propensity scores in patients with complete revascularization. ES = effect size; KS = Komogorov-Smirnov. Figure S3. Standardized effect size plot for estimating propensity scores in patients with incomplete revascularization. ES = effect size; KS = Komogorov-Smirnov. Table S1. Comparison before and after IPTW adjustment for all patients. Table S2. Comparison before and after IPTW adjustment for the CR group. Table S3. Comparison before and after IPTW adjustment for the IR group.
