# Supplementary material for: Incidence, Predictive Factors and Long-Term Clinical Impact of Left Ventricular Remodeling According to the Completeness of Revascularization in Patients with ST-Elevation Myocardial Infarction and Multivessel Disease
Source: J Clin Med. 2022 Oct 23;11(21):6252. doi: 10.3390/jcm11216252 (PMC9656271; doi:10.3390/jcm11216252)
Supplement: Supplementary file 1 [file jcm-11-06252-s001.zip › jcm-1932456-supplementary.pdf]

Supplementary Material

Supplementary Figures

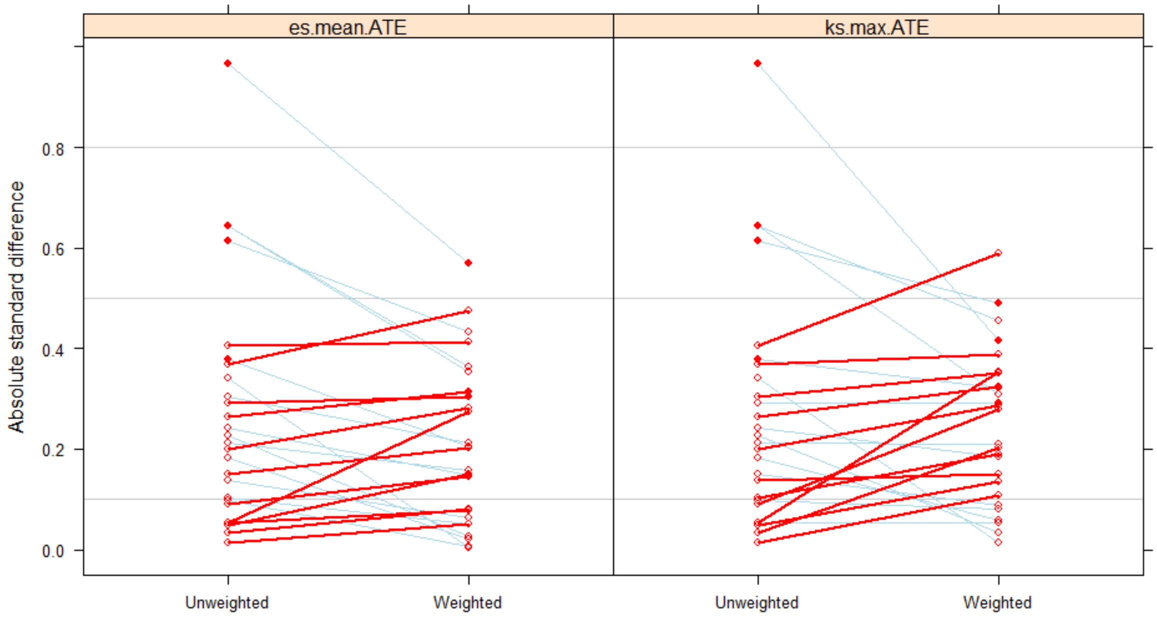

Figure S1. Standardized effect size plot for estimating propensity scores in all patients. ES=effect size; KS=Komogorov-Smirnov.

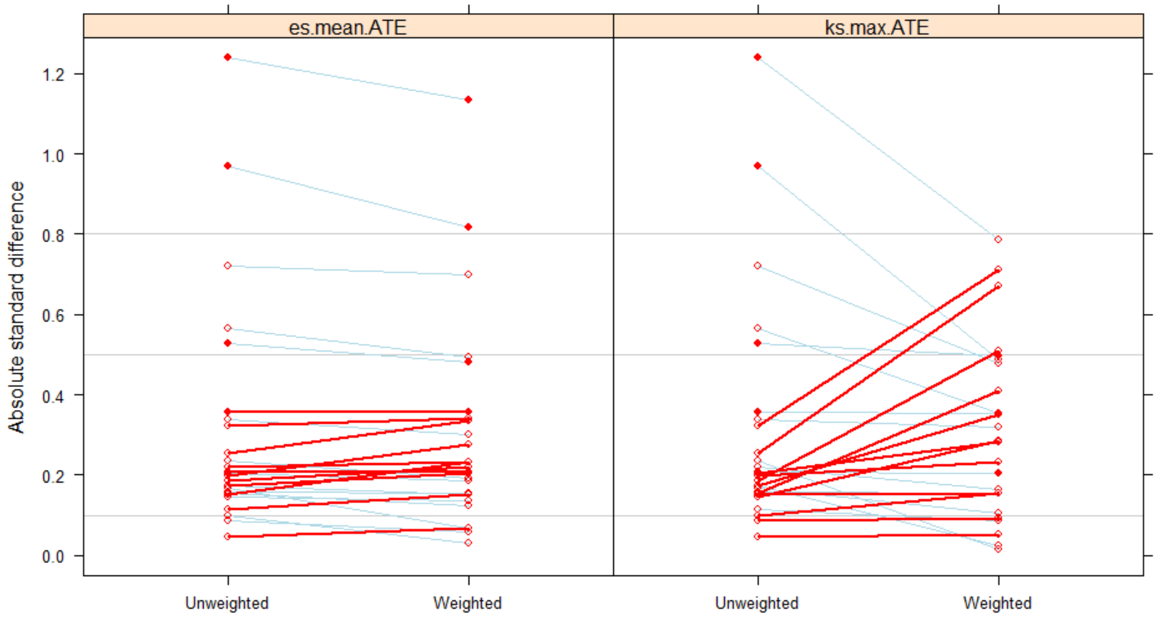

Figure S2. Standardized effect size plot for estimating propensity scores in patients with complete revascularization. ES=effect size; KS=Komogorov-Smirnov.

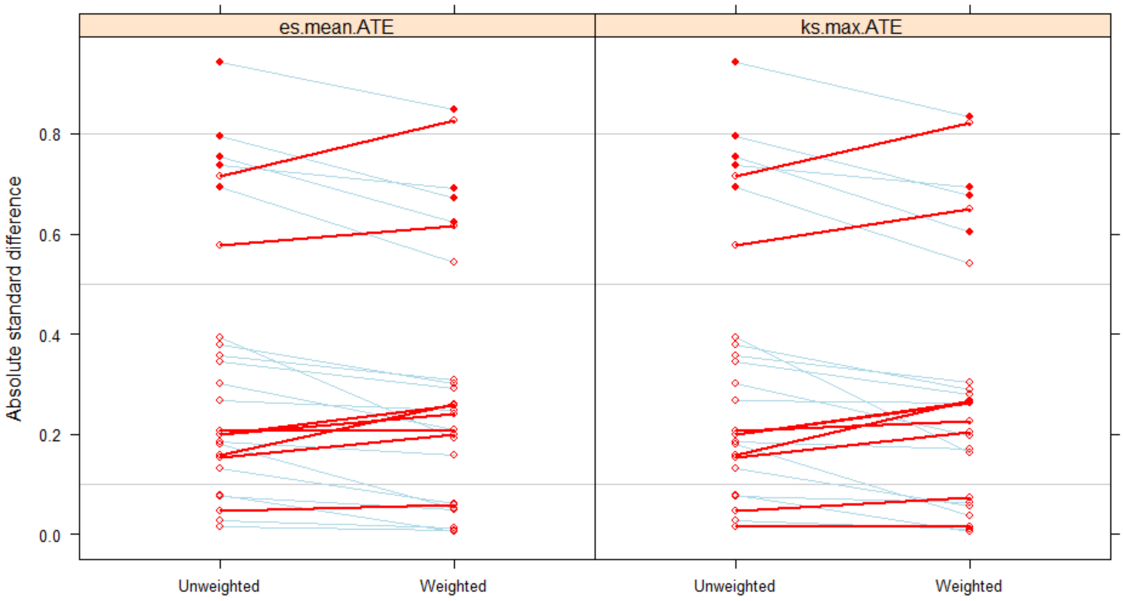

Figure S3. Standardized effect size plot for estimating propensity scores in patients with incomplete revascularization. ES=effect size; KS=Komogorov-Smirnov.

Table S1. Comparison before and after IPTW adjustment for all patients

	Before IPTW adjustment						After IPTW adjustment					
	LV remodeling		No LV remodeling				LV remodeling		No LV remodeling			
	Mean	SD	Mean	SD	SES	P value	Mean	SD	Mean	SD	SES	P value
Age	71.5	10.053	68.047	11.946	0.293	0.099	72.269	7.591	68.13	11.902	0.351	0.007
Male	0.692	0.471	0.733	0.443	-0.091	0.669	0.536	0.509	0.733	0.443	-0.444	0.154
SBP	130.385	23.406	134.915	22.493	-0.201	0.34	132.52	20.729	134.852	22.507	-0.103	0.671
DBP	81.154	13.365	83.898	12.918	-0.212	0.312	83.208	11.078	83.858	12.877	-0.05	0.781
HR	78.692	15.115	73.987	13.567	0.342	0.123	71.026	16.05	74.115	13.612	-0.224	0.488
Killip 2	0.038	0.196	0.123	0.329	-0.265	0.053	0.017	0.131	0.123	0.329	-0.334	<0.001
Killip 3	0.192	0.402	0.042	0.202	0.644	0.057	0.107	0.315	0.045	0.209	0.264	0.279

Previous CAD	0.115	0.326	0.068	0.252	0.183	0.464	0.061	0.245	0.067	0.251	-0.023	0.888
Hypertension	0.423	0.504	0.475	0.5	-0.103	0.615	0.628	0.493	0.475	0.5	0.307	0.197
NT-proBNP (pg/mL)	0.423	0.504	0.284	0.452	0.304	0.171	0.386	0.497	0.284	0.452	0.223	0.432
Smoking	0.615	0.496	0.631	0.483	-0.033	0.874	0.583	0.503	0.633	0.483	-0.105	0.703
Previous CVA	0.115	0.326	0.034	0.181	0.406	0.203	0.111	0.32	0.035	0.184	0.378	0.301
Initial LVEF (%)	47.308	10.472	57.792	10.386	-0.967	0	53.531	10.949	57.426	10.58	-0.359	0.178
Initial LVEDD (mm)	51.5	3.501	48.657	4.661	0.614	0	51.193	4.102	48.761	4.666	0.525	0.032
Hemoglobin (mg/dL)	13.327	2.423	12.499	2.783	0.3	0.1	12.68	2.992	12.513	2.778	0.06	0.86
Creatinine (mg/dL)	0.988	0.31	1.029	0.794	-0.054	0.606	0.933	0.28	1.028	0.786	-0.124	0.227
CK-MB (ng/dL)	122.485	108.247	85.3	99.325	0.369	0.09	109.562	115.338	86.791	100.718	0.226	0.439
Peak troponin I (mg/dL)	94.758	91.357	54.726	56.913	0.644	0.027	71.561	73.568	55.833	58.26	0.253	0.28

NTproBNP	461.846	331.998	316.195	275.847	0.512	0.029	390.655	339.069	319.847	277.258	0.249	0.454
LDL cholesterol (mg/dL)	122.846	28.59	126.492	37.461	-0.099	0.546	131.815	26.684	126.535	37.265	0.144	0.461
HDL cholesterol (mg/dL)	41.346	9.798	46.462	13.791	-0.378	0.015	43.771	10.268	46.35	13.752	-0.191	0.321
Hemoglobin A1C (%)	6.812	1.634	6.478	1.127	0.281	0.304	6.45	1.411	6.478	1.12	-0.024	0.918
Statin at discharge	0.769	0.43	0.852	0.356	-0.227	0.338	0.856	0.358	0.853	0.355	0.008	0.968
Beta-blocker at discharge	0.846	0.368	0.89	0.314	-0.137	0.554	0.885	0.326	0.891	0.312	-0.021	0.921
ACE inhibitor or ARB at discharge	0.808	0.402	0.788	0.409	0.048	0.811	0.845	0.37	0.785	0.412	0.147	0.484
Three-vessel disease	0.346	0.485	0.339	0.474	0.015	0.942	0.359	0.489	0.341	0.475	0.038	0.891
ACE=angiotensin converting enzyme; ARB=angiotensin receptor blocker; BNP=brain natriuretic peptide; CAD=coronary artery disease; CVA=cerebrovascular accident; DBP=diastolic blood pressure; HDL=high-density lipoprotein; HR=heart rate; IPTW=inverse probability												

treatment weighting; LDL=low-density lipoprotein; LV=left ventricle; LVEDD=Left ventricular end-diastolic diameter; LVEF=Left ventricular ejection fraction; NT= N terminal; SES=standardized size effect; SD=standard deviation

Table S2. Comparison before and after IPTW adjustment for the CR group

	Before IPTW adjustment						After IPTW adjustment					
	LV remodeling		No LV remodeling				LV remodeling		No LV remodeling			
	Mean	SD	Mean	SD	SES	P value	Mean	SD	Mean	SD	SES	P value
Age	68.769	10.043	66.391	11.705	0.206	0.409	68.532	9.843	66.387	11.713	0.186	0.449
Male	0.692	0.48	0.759	0.429	-0.155	0.617	0.706	0.474	0.76	0.429	-0.124	0.69
SBP	130.769	21.001	135.188	22.516	-0.198	0.459	129.063	19.959	135.244	22.531	-0.277	0.262
DBP	80.769	10.377	83.684	13.454	-0.221	0.334	80.637	10.173	83.713	13.486	-0.233	0.302
HR	70.923	12.1	73.218	12.379	-0.186	0.502	70.509	12.931	73.211	12.343	-0.219	0.494
Killip 3	0.154	0.376	0.023	0.149	0.719	0.197	0.15	0.371	0.022	0.147	0.699	0.207
Hypertension	0.538	0.519	0.451	0.499	0.175	0.549	0.55	0.518	0.448	0.499	0.205	0.492

NT-proBNP (pg/mL)	0.231	0.439	0.308	0.464	-0.168	0.533	0.276	0.465	0.306	0.463	-0.066	0.83
Smoking	0.615	0.506	0.662	0.475	-0.097	0.744	0.646	0.498	0.662	0.475	-0.032	0.915
LVEF initial (%)	48.692	10.078	58.429	9.66	-0.968	0.001	49.918	10.294	58.142	9.797	-0.818	0.007
Initial LVEDD (mm)	51.154	2.911	48.955	4.233	0.527	0.012	51.017	2.847	49.005	4.234	0.482	0.019
Hemoglobin (mg/dL)	11.946	2.883	12.659	2.804	-0.254	0.379	11.702	2.971	12.642	2.81	-0.335	0.285
Creatinine (mg/dL)	0.969	0.315	0.919	0.339	0.15	0.572	0.996	0.331	0.917	0.337	0.233	0.426
CK-MB (ng/dL)	140.269	117.841	84.732	95.008	0.566	0.09	135.888	119.3	87.378	98.928	0.495	0.157
Peak troponin I (mg/dL)	124.768	110.578	50.904	48.035	1.238	0.015	120.254	111.376	52.592	50.138	1.134	0.032
NTproBNP	351.154	324.4	263.887	263.449	0.324	0.332	355.822	330.271	264.157	263.467	0.34	0.338
LDL cholesterol (mg/dL)	134.231	29.598	128.632	39.029	0.146	0.516	133.718	29.954	128.548	38.935	0.135	0.565

HDL cholesterol (mg/dL)	45.769	9.782	47.474	15.462	-0.113	0.563	45.111	10.353	47.375	15.456	-0.151	0.491
Hemoglobin A1C (%)	6.192	1.023	6.62	1.286	-0.338	0.15	6.233	1.089	6.613	1.281	-0.3	0.264
Statin at discharge	0.769	0.439	0.835	0.373	-0.173	0.592	0.777	0.433	0.834	0.374	-0.151	0.644
Beta-blocker at discharge	0.923	0.277	0.91	0.288	0.047	0.865	0.891	0.325	0.91	0.287	-0.069	0.851
ACE inhibitor or ARB at discharge	0.846	0.376	0.812	0.392	0.088	0.748	0.829	0.392	0.807	0.396	0.058	0.85
Three-vessel disease	0.154	0.376	0.256	0.438	-0.235	0.345	0.171	0.392	0.254	0.437	-0.191	0.49

ACE=angiotensin converting enzyme; ARB=angiotensin receptor blocker; BNP=brain natriuretic peptide; CAD=coronary artery disease; CVA=cerebrovascular accident; DBP=diastolic blood pressure; HDL=high-density lipoprotein; HR=heart rate; IPTW=inverse probability treatment weighting; LDL=low-density lipoprotein; LV=left ventricle; LVEDD=Left ventricular end-diastolic diameter; LVEF=Left ventricular ejection fraction; NT= N terminal; SES=standardized size effect; SD=standard deviation

Table S3. Comparison before and after IPTW adjustment for the IR group												
	Before IPTW adjustment						After IPTW adjustment					
	LV remodeling		No LV remodeling				LV remodeling		No LV remodeling			
	Mean	SD	Mean	SD	SES	P value	Mean	SD	Mean	SD	SES	P value
Age	74.231	9.671	70.184	11.97	0.344	0.158	73.639	8.788	70.208	11.885	0.292	0.171
Male	0.692	0.48	0.699	0.461	-0.015	0.961	0.697	0.478	0.701	0.46	-0.008	0.978
SBP	130	26.458	134.563	22.569	-0.199	0.54	129.161	24.224	134.703	22.631	-0.241	0.401
DBP	81.538	16.251	84.175	12.249	-0.208	0.56	81.607	15.699	84.254	12.271	-0.208	0.554
HR	86.462	14.099	74.981	14.967	0.753	0.005	84.805	14.214	75.29	15.221	0.624	0.025
Killip 2	0.077	0.277	0.146	0.354	-0.198	0.404	0.057	0.241	0.146	0.355	-0.258	0.181
Killip 3	0.231	0.439	0.068	0.253	0.578	0.177	0.243	0.446	0.07	0.256	0.615	0.181

Previous CAD	0.231	0.439	0.107	0.31	0.379	0.309	0.206	0.421	0.108	0.312	0.3	0.393
Hypertension	0.308	0.48	0.505	0.502	-0.393	0.155	0.409	0.512	0.506	0.502	-0.192	0.542
NT-proBNP (pg/mL)	401.308	398.755	360.612	296.972	0.132	0.714	376.876	380.815	357.828	297.154	0.062	0.856
Smoking	0.615	0.506	0.592	0.494	0.047	0.873	0.565	0.516	0.594	0.494	-0.059	0.852
Previous CVA	0.231	0.439	0.049	0.216	0.716	0.129	0.259	0.456	0.049	0.216	0.827	0.127
LVEF initial (%)	45.923	11.079	56.971	11.25	-0.943	0.001	46.765	10.663	56.716	11.387	-0.849	0.001
Initial LVEDD (mm)	51.846	4.1	48.272	5.157	0.693	0.004	51.167	4.968	48.362	5.166	0.544	0.108
Hemoglobin (mg/dL)	12.962	2.686	12.525	2.763	0.159	0.571	13.233	2.592	12.52	2.754	0.259	0.335
Creatinine (mg/dL)	1.008	0.317	1.172	1.127	-0.154	0.243	0.956	0.299	1.168	1.111	-0.199	0.114
CK-MB (ng/dL)	104.7	99.194	86.035	105.103	0.179	0.514	92.196	91.466	86.76	105.58	0.052	0.83

Peak troponin I (mg/dL)	64.749	56.65	59.661	66.58	0.078	0.759	60.176	51.027	59.851	66.428	0.005	0.982
NTproBNP	401.308	398.755	360.612	296.972	0.132	0.714	376.876	380.815	357.828	297.154	0.062	0.856
LDL cholesterol (mg/dL)	111.462	23.369	123.728	35.33	-0.357	0.089	112.915	22.247	123.457	35.233	-0.307	0.122
HDL cholesterol (mg/dL)	36.923	7.868	45.155	11.213	-0.737	0.001	37.255	7.263	44.983	11.196	-0.692	0
Hemoglobin A1C (%)	6.562	1.129	6.476	1.127	0.076	0.79	6.539	1.156	6.485	1.128	0.048	0.877
Statin at discharge	0.769	0.439	0.874	0.334	-0.302	0.393	0.805	0.412	0.878	0.329	-0.208	0.514
Beta-blocker at discharge	0.769	0.439	0.864	0.344	-0.267	0.439	0.778	0.433	0.866	0.343	-0.247	0.484
ACE inhibitor or ARB at discharge	0.769	0.439	0.757	0.431	0.028	0.924	0.748	0.452	0.753	0.433	-0.012	0.972
Three-vessel disease	0.538	0.519	0.447	0.5	0.184	0.534	0.528	0.52	0.448	0.5	0.159	0.607

ACE=angiotensin converting enzyme; ARB=angiotensin receptor blocker; BNP=brain natriuretic peptide; CAD=coronary artery disease; CVA=cerebrovascular accident; DBP=diastolic blood pressure; HDL=high-density lipoprotein; HR=heart rate; IPTW=inverse probability

treatment weighting; LDL=low-density lipoprotein; LV=left ventricle; LVEDD=Left ventricular end-diastolic diameter; LVEF=Left ventricular ejection fraction; NT= N terminal; SES=standardized size effect; SD=standard deviation
